# Discussing the influence of electrode location in the result of esophageal prolonged pH monitoring

**DOI:** 10.1186/1471-230X-14-64

**Published:** 2014-04-04

**Authors:** Valter Nilton Felix, Ioshiaki Yogi, Daniel Senday, Fernando Tadeu Vannucci Coimbra, David Pares, Vinicius Garcia, Carlos Eduardo Garcia

**Affiliations:** 1 Digestive Surgery Division, São Paulo University, and Head of the Nucleus of General and Specialized Surgery, São Paulo, Brazil; 2 Nucleus of General and Specialized Surgery, São Paulo, Brazil; 3 School of Medicine, São Paulo University, R. Frei Caneca, 1407 – cj 221, São Paulo, SP, Brazil

**Keywords:** Gerd, Esophageal ph monitoring, Ph-metric electrode location

## Abstract

**Background:**

There is a large consensus to preserve the distance of 5 cm above the proximal border of the lower esophageal sphincter (PBLES) as appropriate to the location of the electrode of the pH-metry. The main objective of this study is to determine whether placement of the electrode below the recommended location achieves a significant difference in the calculation of the DeMeester score.

**Methods:**

The study was made up of 60 GERD patients and 20 control subjects. They were submitted to esophageal manometry and to pH-metric examination with two pH-metric catheters contained antimony electrodes - the distal was positioned 3 cm above the PBLES, leaving the other 5 cm away from it.

**Results:**

LES pressure (LESP) in the GERD group was significantly lower than in the control group (P = 0.005). Normal mean DeMeester score was observed simultaneously in the control group, by both the electrodes, but abnormal DeMeester score was much more expressive when observed by the distal electrode in the GERD group. There were significant differences as for DeMeester score, of patients with GERD from that of the control group and of distal from the proximal electrode in the GERD group.

**Conclusions:**

Acid reflux is directly related to lower levels of LESP. Lower location of the catheter may strongly affect the results of prolonged esophageal pH monitoring in GERD patients.

## Background

Gastroesophageal reflux disease (GERD) is a multifaceted disease, defined as chronic symptoms or mucosal damage produced by the abnormal reflux of gastric contents into the esophagus and close to 50% of the population will have some type of GERD symptom during a calendar year. In the patient presenting with heartburn and/or regurgitation, the diagnosis of GERD is highly likely and a reference standard of pH monitoring and/or endoscopy has been used to establish a diagnosis of GERD [1].

Endoscopy is the single best test to answer the question of whether mucosal injury or BE (Barrett esophagus) is present [2], but the overall sensitivity of endoscopy for the diagnosis of GERD is less than 50%; that is, less than half of GERD patients will have esophagitis at the time of endoscopy.

Ambulatory esophageal pH monitoring documents the pattern, frequency, and duration of esophageal acid exposure and allows correlation between reflux events and symptoms; thus it is the best test to answer the question: are these symptoms due to reflux of acid? [3]. Early studies found sensitivity and specificity of this test to be extremely high (over 90% sensitive and specific) [4], although other studies, particularly in endoscopy-negative patients, report lower accuracy of the pH-metry [5].

In 1969 the idea of a prolonged intra-esophageal pH measurement (18 hours) [6] was introduced. It was later standardized to a 24 h test [7], with the intention of obtaining data that quantitatively expresses the gastro esophageal reflux (GER), the ability to identify esophageal clearance, observing the duration of each reflux episode, and mimic the acid perfusion test of the esophagus, since the reflux episodes could be correlated with the patient's symptoms, indicated on the graphic by the touch of a specific button in the registration equipment and reported in the journal of the examination.

The possible interference of the presence of the electrode in the esophageal lumen, increasing saliva production and frequency of contractions of the organ was discarded by a classic project [8], while another [9] removed any doubt as to the possibility of electromagnetic interference altering the final result of the exam.

Then the hypothesis of possible food flows, outside of the main meals, clearly marked on the charts, being able to alter significantly the total time of pH less than 4 was rejected [10]. The use of pH 4 as the threshold of normality was established in 1987 [8] and DeMeester score began to be used widely.

As for the positioning of the electrode 5 cm above the proximal border of lower esophageal sphincter (PBLES), as made by Johnson & DeMeester [7] to define the normal exam, with scores less than 14.72, there were always disputes. Some authors prefer to measure it at a distance of 3 cm [11], while others put the lower limit of LES as a starting point for the measurement of 5 cm [12].

These changes were not well received, although some occasional question as for the classic positioning of the electrode remains, for example arguing that if the reflux did not reach that level it could go unnoticed, even when causing GERD complicated by the emergence of Barrett's epithelium in the more distal esophagus [13].

However, there is a large consensus both in Brazil [14] and in the exterior [15] to continue preserving the distance of 5 cm above the upper border of the LES, preferably located by prior esophageal manometry, as appropriate to the location of the electrode of the pH-metry.

The main objective of this study is to determine whether placement of the electrode, below the recommended location, achieves a significant difference in the calculation of the DeMeester score. The secondary objective includes verifying correlations of pH monitoring and LES pressure in GERD.

## Methods

The study was made up of 60 patients (28 men and 32 women, average age: 39.29 ± 10.04 years), with BMI never exceeding 25 Kg/m^2^, who were all suffering from a heartburn complaint, presenting episodical extra-esophageal symptoms, in which digestive endoscopy previously identified erosive esophagitis Los Angeles B without hiatal hernia. Clinical and endoscopic criteria, after a minimum period of one year under unsuccessful heartburn clinical control tentative including PPI, procinetics, postural and habits orientation, established indication for surgery. Incomplete control of symptoms or almost immediate recurrence after suspension of treatment occurred even using PPI at a dose of 80 mg/day. Twenty control subjects (8 men and 12 women, mean age: 43.3 ± 11.50 years), volunteers, without esophageal complaints, with normal esophagoscopy, were submitted to the same study protocol.

All subjects signed a consent form following the standards of the Helsinki Convention. This study was approved by the ethics committee of São Paulo – Rio Preto Faculty of Medicine.

The first procedure was esophageal manometry. With the patients in semi-recumbent position, the catheter was pulled out by 1 cm steps, crossing the high pressure zone of the gastro-esophageal junction. This procedure was preceded by suspension of all medicines prescribed for palliation of peptic symptoms for 72 hours and fasting for 8 hours, and followed by the installation of two pH-metric catheters through one nostril with the patient in a sitting position.

The catheters contained antimony electrodes and, after appropriate calibration, the distal was positioned 3 cm above PBLES, leaving the other 5 cm away from that anatomical and functional reference.

From then on the procedure followed the usual pattern of the pH-metric examination. The patients were instructed to keep their habitual daily activities and to record food and fluid consumption, and posture changes, on a diary card. Two Digitrapper Mark III pH recorder Synectics, Medtronics® were employed and after about 24 hours the data collected in the register were translated and processed in the Synectics® program, each electrode providing an independent database. Acid reflux was defined as a sudden drop in esophageal pH below 4.

With the processed data, a search was made to determine:

• The length and resting pressure of LES (LESP) obtained from the average of the records of the four distal circumferential manometric sensors, and the location of PBLES, in patients and control subjects;

• If there were significant differences between the mean length and resting pressure of the LES in both groups;

• The DeMeester scores corresponding to the data collected by the electrodes positioned 3 cm and 5 cm from PBLES and if there was a significant difference between their averages, enabling an intra and intergroup analysis.

The data were subjected to statistical analyzes, using the Student *t* test to compare quantitative data, accepting P ≤ 0.05, with a confidence limit of 95%.

## Results

No patient or control had an extension of LES less than 3 cm, always with at least 1 cm located below the diaphragm, while the average LESP in the group of patients with GERD (12.24 ± 7.02 mmHg) was significantly lower (P = 0.005) than that noted in the control group (18.4 ± 7.76 mmHg) (Table 1).

**Table 1:** Averages of extension (EXT) and resting pressure of the lower esophageal sphincter (LESP)

**Group**	**EXT (cm)**	**LESP (mmHg)**
GERD	3.61 ± 0.38	12.24 ± 7.02
Control	3.97 ± 0.49	18.4 ± 7.76
P	0.622	0.005

No discrepancy with respect to DeMeester score was observed, when considering the results obtained simultaneously in the control group, by the electrodes situated 3 cm and 5 cm from PBLES, normal in both and without statistical difference between averages (11.27 ± 1.69 and 10.10 ± 2.33) (P = 0,37). The mean scores corresponding to the data captured by electrodes placed 3 cm and 5 cm from PBLES in patients with GERD (respectively, 25.67 ± 12.23 and 15,87 ± 11.71) were significant different from one to other (P = 0.008) (Table 2).

**Table 2:** DeMeester score for different levels of the pH-metry probe above the proximal border of the lower esophageal sphincter (PBLES)

**GROUP**	**3 cm above PBLES**	**5 cm above PBLES**	**P**
GERD	25.67 ± 12.23	15.87 ± 11.71	0.008
CONTROL	11.27 ± 1.69	10.10 ± 2.33	0.37
P	0.001	0.022	

Both for the electrode positioned 3 cm, and for the one located 5 cm from PBLES, there were significant differences as to the DeMeester score, of patients with GERD from that of the control subjects (respectively P = 0.001 and P = 0.022) (Table 2).

The catheter located 5 cm above the PBLES registered a normal DeMeester score in 26/60 patients (43.33%) and in 20/20 control subjects, implying sensitivity of 56.66% and specificity of 100% of conventional prolonged esophageal pH monitoring. The catheter located 3 cm above PBLES increased sensibility to 80%, upholding specificity of 100% (from normal DeMeester score in 12/60 (20%) patients and in 20/20 control subjects).

## Discussion

Ambulatory esophageal pH monitoring is classically performed by placing a pH electrode 5 cm above the proximal border of the lower esophageal sphincter [3]. The most useful parameter for documentation of pathologic reflux is the DeMeester score, but the test has its limitations, with reported sensitivities ranging from 60% to 100% [16] through to indexes as low as 28% [17].

Ferdinandis et al. [18] found pathological acid reflux in 43 patients (31%) at the esophageal pH monitoring, helping to establish a cause for the morbidity in a significant number of patients with GERD symptoms, but not in the majority of patients referred for the test.

The inconstant sensibility of the exam can make its methodology doubtful and then some points need to be considered if it´s normal:

• A non-reflux diagnosis, such as achalasia, gastroparesis or functional heartburn, or non-acid reflux. Currently available technology, such as impedance monitoring, bilimetry, esophageal manometry and/or gastric scintigraphy, might help us to identify many patients who have non-reflux disease or non-acid reflux [19,20];

• The patients could have missed acid reflux that was not picked up on a single day study: 25% of cases monitored by capsule pH testing could have normal findings one day and abnormal findings the next day in a 48 h study [21]. However, Hakanson et al. [22] report that no studies were cited in the published guidelines that indicate superior outcomes for patients for treatment guided by wireless pH testing versus traditional pH testing. The major advantage for the wireless system cited was patient tolerability;

• The pH probe might have missed distal acid reflux [13];

• Alternatively, noxious effect of the nasal catheter could have limited both eating and activity and resulted in a false-negative test.

The latter possibility could perhaps be overcome with better explanation of the background of the examination to the patients, giving them greater involvement and ensuring their cooperation, thus resulting in more effective testing.

Traditionally, the pH probe is placed 5 cm above the proximal border of the lower esophageal sphincter. One study found that over a period of 24 h, the amount of acid exposure in 11 endoscopy-negative dyspeptic patients was greater if measured 5 mm above the squamo-columnar junction than when measured at the conventional 5 cm above the squamo-columnar junction (11.7% vs 1.8%; P <0.001) [23]. These authors suggested a lower placement of the electrode to better detect the gastro-esophageal reflux.

The problem, however, is how much this reflux can be aggressive to the region most distal of the esophagus, which may have increased resistance and much faster clearance. This without mentioning the fact that drastic change in catheter placement requires new standards of quantitative interpretation of the examination.

This was the starting point of this study, considering that the validity of pH monitoring scores is currently linked to the positioning of the electrode 5 cm from PBLES. Therefore, it is necessary to discuss whether a slight change that would alter this methodological aspect interferes with the result of the examination, taking care to use manometric equipment and esophageal pH monitoring, as well as catheters with a recognized technical reliability.

We excluded patients with hiatal hernia to avoid distortion of the results due to the mobility of esophagogastric junction, inherent to that anatomical condition.

The results showed that electrodes located either 3 or 5 cm from PBLES show similar normal DeMeester scores, in the control group, reflecting that, in fact, as previously reported [24], placing the electrode slightly below the conventional 5 cm from PBLES tends not to alter the pH-metric final result in normal subjects. However, in patients with GERD, there is a significant difference between the averages obtained by the electrodes placed 3 cm or 5 cm above PBLES, much more reflux being registered by the distal electrode, although abnormal averages have been observed in both.

LESP may not be the only determinant factor in LES competency, but it could be of great importance. DeMeester, using three criteria (LES hypotony, intra-abdominal sphincter length < 1 cm or total sphincter length < 2 cm), found that there was a 70% chance of abnormal reflux if one of the three above factors was present. If all three criteria were met, reflux was seen in 92% of patients [25].

In this series a reduction in length of the LES in patients or control subjects was not observed, but resting sphincter pressure, in fact, proved be directly related to reflux, so that the average LESP in the group of patients with GERD (12.24 ± 7.02) was significantly lower (P = 0.005) than that noted in the control group (18.4 ± 7.76 mmHg).

Particularly among normal individuals, LESP seems to provide a so good anti-reflux protection that normal DeMeester score can be observed even with the electrode positioned 3 cm above the PBLES. On the other hand, in GERD patients, its lower value can determine significant difference between distal and proximal electrodes measurements. Therefore it can be observed that acid reflux is directly related to lower levels of resting sphincter pressure, and that sensibility of the conventional prolonged esophageal pH monitoring could be increased with lower location of the pH-metric electrode. Charbel et al. [26] must be recollected, because they stated that pH monitoring is most likely to be normal using conventional and the most stringent methodological criteria [26].

## Conclusions

Placement of the electrode below the recommended location could be harmful in some GERD patients, simulating a much more severe gastroesophageal reflux, maybe implying unnecessary fundoplicature. However we could eventually underestimate the reflux locating the catheter at 5 cm, too far from the PBLES. New studies could help to clarify this doubtful and important matter.

### Helsinki statement

All subjects signed a consent form, after receiving detailed informations about all aspects of the work and about the observation of the standards of the Helsinki Convention.

## Abbreviations

GERD: Gastroesophageal reflux disease; GER: Gastroesophageal reflux; PBLES: Proximal border of lower esophageal sphincter; LESP: Resting pressure of lower esophageal sphincter.

## Competing interests

The authors declare that they have no competing interests.

## Authors' contributions

VNF conceived of the study and drafted the manuscript. VNF, IY, DS, FTVC and DP carried out the exams. DP, VNG and CEG participated in the design of the study and performed the statistical analysis. IY, DS and FTVC participated in its design and coordination. All authors read and approved the final manuscript.

## Pre-publication history

The pre-publication history for this paper can be accessed here:

http://www.biomedcentral.com/1471-230X/14/64/prepub
